# The impact of scanning data measurements on the Acuros dose calculation algorithm configuration

**DOI:** 10.1186/s13014-020-01610-7

**Published:** 2020-07-10

**Authors:** A. Fogliata, E. Esposito, L. Paganini, G. Reggiori, S. Tomatis, M. Scorsetti, L. Cozzi

**Affiliations:** 1 Radiotherapy and Radiosurgery Dept, Humanitas Clinical and Research Center – IRCCS, Via Manzoni 56, Milan-Rozzano, Italy; 2 Department of Biomedical Sciences, Humanitas University, Via Rita Levi Montalcini 4, Milan-Pieve Emanuele, Italy

## Abstract

**Background:**

Many dose calculation algorithms for radiotherapy planning need to be configured for each clinical beam using pre-defined measurements. An optimization process adjusts the physical parameters able to estimate the energy released in the medium in any geometrical condition. This work investigates the impact of measured input data quality on the configuration of the type “c” Acuros-XB dose calculation algorithm in the Eclipse (Varian Medical Systems) treatment planning system.

**Methods:**

Different datasets were acquired with the BeamScan water phantom (PTW) to configure 6 MV beams, for both flattened (6X) and flattening filter free mode (6FFF) for a Varian TrueBeam: *(i)* a correct dataset measured using a Semiflex-3D ion chamber, *(ii)* a set in missing lateral scatter conditions (MLS), *(iii)* a set with incorrect effective point of measurement (EPoM), *(iv)* sets acquired with PinPoint-3D chamber, DiodeP, microDiamond detectors.

The Acuros-XB dose calculation algorithm (version 15.6) was configured using the reference dataset, the sets measured with the different detectors, with intentional errors, and using the representative beam data (RBD) made available by the vendor. The physical parameters obtained from each optimization process (spectrum, mean radial energy, electron contamination), were analyzed and compared. Calculated data were finally compared against the input and reference measurements.

**Results:**

Concerning the physical parameters, the configurations presenting the largest differences were the MLS conditions (mean radial energy) and the incorrect EPoM (electron contamination). The calculation doses relative to the input data present low accuracy, with mean differences > 2% in some conditions. The PinPoint-3D ion chamber presented lower accuracy for the 6FFF beam. Regarding the RBD, calculations compared well with the input data used for the configuration, but not with the reference data.

**Conclusion:**

The MLS conditions and the incorrect setting of the EPoM lead to erroneous configurations and should be avoided. The choice of an appropriate detector is important. Whenever the representative beam data is used, a careful check under more clinical geometrical conditions is advised.

## Introduction

In the radiotherapy process, the accuracy of the dose distribution calculation is fundamental. The requirements for the dose delivery accuracy have been based, over the years, on the probability of tumor control (TCP) and normal tissue complications (NTCP), which are sigmoidal curves. Uncertainty in the dose delivery can easily translate in a reduction of the TCP, or an increased NTCP relative to expectations, leading to a worsened patient outcome. In the 1980s, Brahme [1, 2] evaluated a variation of 6–7% in NTCP for a 1% change in delivered dose. It has already been pointed out in the ICRU Report 24 [3] that in critical situations, a dose calculation accuracy of ±5% must be achieved, but ±2% may be required. A figure of ±3% was later recommended by Brahme [2]. In 1993, Van Dyk et al. [4] suggested acceptability criteria for photon beams, with ±2% as an achievable goal for dose calculation algorithms accuracy. Acceptable uncertainty levels include many steps (and related uncertainties) of the dose calculation and delivery: *(i)* the accuracy of the beam configuration, *(ii)* the accuracy of the dose calculation in the patient including all the anatomical and density variations as well as the complexities derived from the modern techniques of intensity modulation, *(iii)* the accuracy in the beam calibration, etc.

Dose calculation algorithms are today classified in three categories [5, 6], type “a”, “b”, “c”, according to their handling of managing the lateral electron transport. Type “a” includes the pencil beam algorithm (no lateral electron transport); type “b” comprises algorithms like the collapsed-cone or the anisotropic analytical algorithm AAA (the lateral electron transport is modeled); type “c” embraces the Monte Carlo and Boltzmann Transport equation solvers, like Acuros-XB (Acuros in the following). Any dose calculation algorithm derives its accuracy also from the accuracy of the beam modeling, which collects in different ways the information on the beam generated by the linear accelerator, the head scatter, and all the radiation components that contribute to the beam generation. The beam modeling step is a crucial point, also for the algorithms (type “c”) that are the most accurate in dose estimation in heterogeneous media [6–9].

Most commercial treatment planning systems have to be commissioned for each beam generated by a clinical linear accelerator, according to the vendor’s procedures optimizing several parameters, based on a predefined set of measured data. The quality of the beam configuration should be as good as possible, since this step is only one of the steps affecting the total uncertainty of the dose delivery. Of course, the input measured data should be of high quality to obtain the best agreement between the actual clinical beam and the beam modeled in the system.

In recent years, the use of pre-acquired beam data (golden beam data, or representative beam data) for modeling the clinical beam, has started to be more and more used in clinical practice. However, the need to evaluate the dose calculation algorithm quality against the specific clinical beam remains.

The dose calculation accuracy is the ability of the algorithm to estimate the dose distribution (energy deposition) in the medium [5, 9–15]. However, there is a lack of studies evaluating the accuracy of the implementation of an algorithm in the planning system, except in the specific case of small field, where a lot of publications describe in detail the accuracy and the tuning of specific parameters [14, 16–21].

The aim of this work is to evaluate the impact of the scanning measurement quality (profiles and depth doses) on the configuration process for the type “c” Acuros dose calculation algorithm implemented in the Eclipse treatment planning system (Varian Medical Systems, Palo Alto, USA), focusing on possible (or typical) errors that may occur during the acquisition of the commissioning measurements. Also, the use of the representative beam data is analyzed.

## Materials and methods

Different measurements, with and without intentional measurement errors, were acquired to configure the Acuros dose calculation algorithm, in the Eclipse treatment planning system, version 15.6.03, for a 6 MV and a 6 MV flattening filter free FFF beam from a TrueBeam linac (Varian Medical System, Palo Alto, USA).

### Required measurements

The Acuros configuration process requires a minimum set of measurements that need to be acquired on the linac. This includes:

- depth dose curves (PDD) for a number of square field sizes, at a defined source to phantom distance (SSD). The field sizes should include MINxMIN, AxA, 10 × 10, BxB, MAXxMAX cm^2^, where MIN is the smallest, typically 3 × 3 cm^2^, A is between the smallest and 10, B between 10 and MAX, which is the largest;

- profiles on one of the main axes, at d_max_, 5, 10, 20, 30 cm depth for the same field sizes and SSD as the PDD. Profiles tails outside the field are required to a distance of at least 3.5 cm.

- diagonal profiles for the maximum field size, at the same depths and SSD as the other profiles with the collimator set to 45 degrees, so that the profiles are acquired along the same axis (relative to the beam and not the collimating device) as the other profiles.

- output factors for a number of square and rectangular field sizes. SSD can be different from that used for scanning data, depth has to be larger than d_max_, suggested 5 or 10 cm.

- MLC data regarding the transmission and dosimetric leaf gap are needed.

The beam model configuration is based on fields shaped by the jaws. The MLC parameters, which are fundamental in the IMRT or VMAT commissioning, do not influence the results of the beam configuration, while they need to be adjusted for MLC field handling. Details and adjustment of those parameters are outside the scope of this work, and their tuning is already the subject of some publications [17, 18].

### Measurements

Data were measured on a Varian TrueBeam linac, equipped with a Millennium 120-MLC, for the energies of 6 MV with flattening filter (6X in the following), and 6 MV without flattening filter (6FFF in the following). No wedged beams were measured.

The set of data acquired in different conditions for the current work included:

- PDD for 3 × 3, 6 × 6, 10 × 10, 15 × 15, 20 × 20, 40 × 40 cm^2^ at SSD = 90 cm.

- profiles for the same fields and SSD, at the required depths of 1.5 (d_max_), 5, 10, 20, 30 cm.

- diagonal profiles according to requirements.

- output factors at 10 cm depth, isocentre (SSD = 90 cm) for the following field sizes: 2, 3, 4, 6, 8, 10, 12, 15, 20, 30, 40 cm side.

For all the acquisitions, a BeamScan water phantom (PTW, Freiburg, Germany) with automatic leveling and alignment was used. All the acquired data have been smoothed, the profiles re-centered (when possible) and symmetrized using the tools in the Mephysto software (PTW, Freiburg, Germany), version 4.3.1, to have an optimal and controlled dataset to input in the beam configuration process.

Different situations were considered to evaluate the impact of possible measurement errors: *(i)* missing lateral scatter (MLS) during the half-beam (or incomplete profile) acquisitions in water tank not large enough to entirely accommodate the beam, *(ii)* the vertical direction scanning origin with incorrect effective point of measurement (EPoM) setting, *(iii)* different detector choices.

- *Reference set*: This dataset was considered as the reference and was acquired with a Semiflex-3D ion chamber, type 31021 (PTW, Germany), positioned with the stem vertical to ensure the same detector orientation for all fields, for all the acquisitions, both scanning (PDD and profiles), and point measurements (output factors). The nominal sensitive volume of the Semiflex-3D is 0.07 cm^3^, with a radius of 2.4 mm and a length of 4.8 mm. For the largest fields, and especially for the diagonal profiles, particular attention was paid to have acquisitions in conditions of full lateral scatter (with the whole field area irradiating the phantom, or to the largest possible). This dataset was considered to be the reference. The output factors of this dataset were used for all configurations.

- *Missing lateral scatter:* the water tank was positioned with the beam central axis at only 7 cm from a phantom lateral wall, to force missing lateral scatter. All scanning data were acquired in those conditions with the Semiflex-3D ion chamber; the beam area for fields larger than 15 × 15 cm^2^ was partially out of the phantom.

- *PDD ± 3 mm, PDD_centreIC:* for these settings no extra measurements were acquired. The depth coordinates of the PDDs were shifted by 3 mm toward the source (PDD-3 mm) or the phantom (PDD + 3 mm), or only 1.5 mm more in depth. The choice of 3 mm was considered as a possible error in setting the effective point of measurements, EPoM (about twice the shift to apply from the central axis toward the source for the EPoM, or from the chamber surface downward), while the 1.5 mm intended to mimic the condition of a Semiflex ion chamber positioned with the zero at the chamber axis when horizontally positioned, i.e. without correcting for the EPoM.

- *DiagonalSSD70cm:* the diagonal profiles only were acquired with a Semiflex-3D in the same conditions as above, but with an SSD equal to 70 cm. The profiles were then geometrically rescaled to an SSD of 90 cm. The other profiles and PDD of this dataset were the same as the Reference set. This situation is intended to mimic a different solution to accommodate large profiles in the water tank.

- *PinPoint-3D:* The same scanning measurements (PDDs, profiles, and diagonals) were acquired in the same conditions as the Reference set, by using the PinPoint-3D ion chamber, type 31022 (PTW, Germany), positioned with stem vertical. The nominal sensitive volume of this detector is 0.016 cm^3^, with a radius of 1.45 mm and a length of 2.9 mm. To ensure the correct positioning of the zero in the vertical direction, the PDD with the Semiflex-3D and PinPoint-3D were made to coincide in the buildup region.

- *DiodeP*: As above, with the scanning measurements acquired with the Diode P detector, type 60016 (PTW, Germany), that is a shielded diode (to correct for diode low energy dose underestimation, making the diode suitable for photon beams) with a nominal sensitive volume of 0.03 mm^3^, with a 1 mm^2^ circular area, 30 μm thick. The entrance window is of 2.42 mm water equivalent thickness. The usability range of this detector is stated by the vendor from 1 × 1 to 40 × 40 cm^2^ field sizes.

- *MicroDiamond*: As above, with the scanning measurements acquired with the microDiamond detector, type 60019 (PTW, Germany), that is a synthetic diamond detector with a nominal sensitive volume of 0.004 mm^3^, with a circular shape of 1.1 mm radius, 1 μm thick. The entrance window is of 1 mm water equivalent thickness. The usability range of this detector is stated by the vendor from 1 × 1 to 40 × 40 cm^2^ field sizes.

### The beam source model

The beam source model generates, from the input measurements, a set of parameters able to modify a parametrized phase space that was obtained from previously published Monte Carlo simulation of linac heads for machines of the same type as the beam under configuration. The model is described in the paper by Tillikainen et al. [22]. This model is implemented in Eclipse for configuring both the AAA (Anisotropic Analytical Algorithm) and Acuros algorithms.

Briefly, the beam source is modeled in three components: the primary photon source, the second photon source, and the electron contamination source.

The primary photon source is a point source located at the target plane (the finite size is modeled by the effective target spot size parameters), and an initial photon spectrum on the beam central axis is given from a library of simulated spectra, tuned to obtain the machine-specific spectrum after the target. The beam hardening in the flattening filter and spectrum variation from the central axis to the field edges is modeled by the mean radial energy curve, obtained from the measured diagonal profiles evaluating the variation in the radial off-axis penetration. Moreover, the non-uniform photon energy fluence is modeled using an intensity profile curve, obtained from the diagonal profile (radial distance) acquired at the shallowest depth (d_max_).

The second source (called extra-focal photon radiation in the Tillikainen et al. study) is modeled as a Gaussian plane source located at the bottom of the flattening filter and describes the head scattering (generated primarily by the flattening filter, and by the primary collimator and secondary collimating devices). It is parametrized as the size of the second source (width of the Gaussian), its weight relative to the primary source, and the mean energy of the second source spectrum. In the model implementation in Eclipse, the second source is disabled for the flattening filter free (FFF) beams.

The electron contamination component describes the dose in the buildup region that is not accounted for in the primary and secondary source components. It hence includes also some photon contamination. It is modeled with a depth-dependent curve. In Acuros the electron contamination is determined by fitting a set of monoenergetic electron depth doses to obtain the electron contamination curve. The electron fluence uses two Gaussian components, the second allowing to model the electron scattering in the air column; their width (σ), as well as the relative fraction of the second component, are estimated in the model configuration.

Finally, for MU calculation, a collimator backscatter factor (CBSF) is determined during the configuration process. This factor takes into account the radiation scattered mainly from the collimating devices (jaws) back to the monitor chamber. This extra radiation is not modeled, while measured by the monitor chamber to count the MU during delivery. The measured output factors are compared to the output factors computed in the model to derive the CBSF table.

### The algorithm configurations and evaluation

The following configurations for Acuros were optimized according to the measurements or their manipulation as described above.

- *Ref:* with the Reference dataset.

- *MLS:* with the entire scanning dataset in conditions of missing lateral scatter.

- *DiagMLS:* with only the diagonal profiles in conditions of missing lateral scatter (profiles and PDD of the Reference dataset).

- *ProfMLS:* with only the profiles in conditions of missing lateral scatter (diagonals and PDD of the Reference dataset).

- *PddMLS:* with only the PDDs in conditions of missing lateral scatter (profiles and diagonals of the Reference dataset).

- *DiagSSD70*: with the diagonal profiles acquired at SSD = 70 cm.

- *PDD + 3 mm:* with the Reference dataset with the PDDs shifted of 3 mm downward.

- *PDD-3 mm:* with the Reference dataset with the PDDs shifted of 3 mm upward.

- *PDD_centreIC:* with the Reference dataset with the PDDs shifted of 1.5 mm downward.

- *PinPoint:* with all the scanning data acquired with the PinPoint-3D ion chamber.

*- DiodeP:* with all the scanning data acquired with the DiodeP detector (only for 6X).

- *microDiamond:* with all the scanning data acquired with the microDiamond detector (only for 6X).

For each configuration, the following parameters generated by the configuration process were analyzed and compared:

- for the primary source:

- the spectrum (as modified by the beam configuration process).

- the mean radial energy.

- the intensity profile.

- for the second source:

the source size, weight, and mean energy parameters.

- for the electron contamination:

the electron contamination depth dose curve.

- the parameters of the two Gaussians.

- for the MU calculation:

the CBSF for square fields, although the same output factors were used as input in all the configurations.

For each configuration, the calculated profiles and PDDs (for the same conditions as the input data) were compared against the input measurements to evaluate the ability of the model to reproduce the data, and against the Reference dataset to estimate the calculation error induced by wrong measurements relative to the (supposed) actual clinical beam. All data were resampled at 1 mm spacing. PDDs were evaluated after the buildup, from 1.8 to 30 cm depth as the difference relative to the point measurement (local percentage difference). Profiles were analyzed in the in-field region (5 mm per side inside the field size rescaled to the acquisition depth) as the difference relative to the point measurement (local percentage difference) and in the region out-of-field, from 5 mm outside the field size rescaled to depth to the furthest measured point. The difference between calculation and measurement was compared, relative to 100% at the beam central axis (global percentage difference).

The analysis was performed outside the beam modeling software, by using a dedicated script in MATLAB R2015a (MatWorks Inc., Natick, MA, USA).

Finally, configuration parameters of the *Ref* were compared with those obtained from the configuration optimized using the representative beam data (RBD) made available to the users by Varian on the myVarian.com platform. The most important differences of the RBD dataset relative to the current work setting were the SSD (100 cm for the RBD) and the detector (cc13 ion chamber (IBA Dosimetry, Schwarzenbruck, Germany) with a sensitive volume of 0.13 cm^3^, 3.05 mm radius and 5.8 mm long).

## Results

PDD measurements for the smallest and largest field size in all the acquisition conditions, as well as profiles at shallow and large depths, are presented in the Supplementary Material of this paper. Also, the calculations for the same curves are shown there for all the configurations.

### Configuration parameters

#### Primary source

**Fig. 1:**
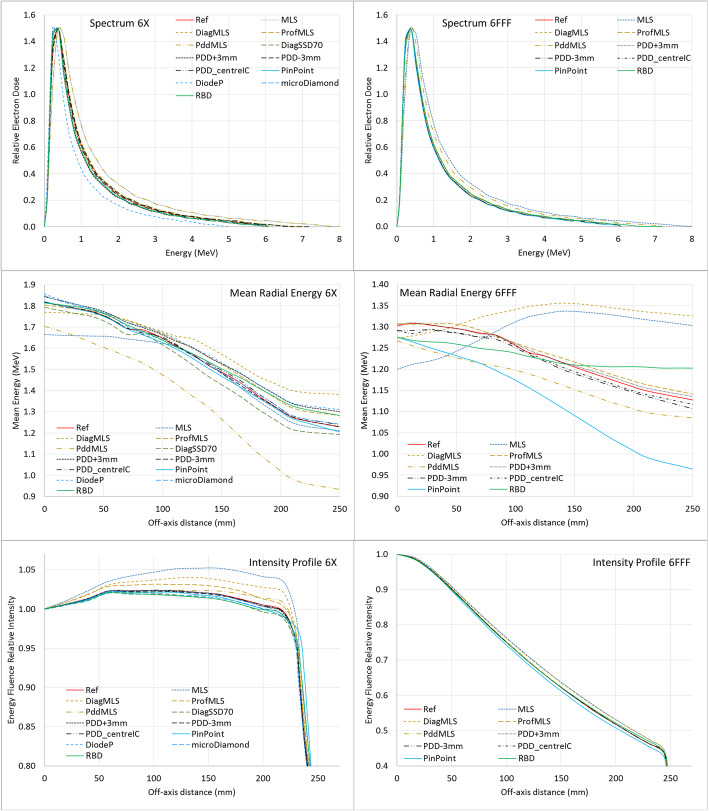
Spectrum after the target, mean radial energy and intensity profile for the 6X (left column) and 6FFF (right column) beams in the different algorithm configurations

Figure 1 reports the primary source parameters: energy spectrum, mean radial energy, and intensity profiles for the different configurations, for both the studied energies, 6X and 6FFF.

The mean energy estimated from the spectra after the target (initial spectrum) and reported in Fig. 1 are summarized in Table 1, together with the mean energy after the flattening filter position, as reported by the mean radial energy on the central axis. For 6X, most of the configurations present similar spectra, except the DiodeP yielding a lower initial energy, and the MLS and Pdd_MLS with a higher initial energy. The mean energy increases going from the target to the bottom of the flattening filter position since part of the low energy photons are absorbed by the high-Z filter material. This rise brings the DiodeP and the MLS to have the highest and lowest energy, respectively, after the filter, consistent with the measurements: the largest field PDD acquired in MLS conditions is less penetrating, while diode presents a higher penetration behavior (Supplementary Material). Noteworthy, the DiodeP mean energy almost doubled after the filter, while the MLS variation was only about 10%, to compare with a mean energy increase of ~ 40% in all the other configurations. For the 6FFF, the mean energy increase is less than 5% (in absence of the filter) for the majority of the configurations, while the MLS presents a mean energy reduction of 10–20%. The unexpected behavior of the MLS energy configuration could suggest an intrinsic error in the configuration process for the missing lateral scatter conditions.

**Table 1:** Configuration parameters

6X	Mean energy [MeV] (after target)	Mean energy [MeV] (on cax, after FF*)	Size of second source [mm]	Relative intensity of second source [%]	Mean energy of second source [MeV]	e-cont σ_0_ at SAD [mm]	e-cont σ_1_ at SAD [mm]	Relative fraction σ_0_
Ref	1.288	1.814	33.6	4.4	0.951	7.930	110.8	0.363
MLS	1.495	1.664	36.9	4.4	0.810	1.001	116.8	0.332
Diag_MLS	1.341	1.770	35.4	4.8	0.941	8.856	105.1	0.315
Prof_MLS	1.288	1.804	35.1	4.6	0.893	8.037	103.1	0.325
Pdd_MLS	1.508	1.704	37.1	3.9	0.828	10.22	119.3	0.245
DiagSSD70	1.331	1.794	30.6	4.2	1.028	8.712	106.1	0.321
PDD + 3 mm	1.207	1.844	33.9	4.1	0.979	350.0	500.0	0.005
PDD_centreIC	1.272	1.821	31.9	4.2	1.006	80.60	116.4	0.314
PDD-3 mm	1.313	1.817	34.3	4.6	0.972	1.018	128.6	0.697
PinPoint	1.229	1.820	30.9	3.1	0.760	1.001	97.4	0.354
DiodeP	0.996	1.858	40.0	5.3	1.066	14.68	103.5	0.240
microDiamond	1.235	1.815	35.9	4.6	1.002	13.30	107.3	0.179
RBD	1.223	1.815	30.2	4.6	1.167	1.001	95.8	0.334
6FFF	Mean energy [MeV] (after target)	Mean energy [MeV] (on cax, after FF*)	Size of second source [mm]	Relative intensity of second source [%]	Mean energy of second source [MeV]	e-cont σ_0_ at SAD [mm]	e-cont σ_1_ at SAD [mm]	Relative fraction σ_0_
Ref	1.266	1.302		0.00		9.559	93.85	0.344
MLS	1.502	1.200		0.00		1.004	108.4	0.540
Diag_MLS	1.306	1.273		0.00		1.002	90.0	0.487
Prof_MLS	1.303	1.306		0.00		1.002	94.8	0.494
PDD_MLS	1.433	1.267		0.00		1.003	104.4	0.450
PDD + 3 mm	1.263	1.307		0.00		349.0	498.8	0.005
PDD-3 mm	1.268	1.291		0.00		1.004	96.02	0.714
PDD_centreIC	1.266	1.292		0.00		19.16	102.1	0.111
PinPoint	1.251	1.275		0.00		1.002	86.4	0.408
RBD	1.313	1.258		0.00		6.648	97.9	0.307

*FF = flattening filter, or foil, in case of FFF beam

The mean radial energy curve, according to the vendor indications, should decrease from the beam central axis to the field edge. The major difference in this parameter is for the MLS and DiagMLS, where for the 6FFF beam an increase is shown instead. This suggests that the variation of the mean radial energy is modeled with the diagonal profiles. The flattening filter yields a beam softening from the central axis to the beam periphery due to the decreasing thickness of the high-Z material filter, resulting in a reduction of the mean radial energy. In the FFF beams, this energy reduction is minimized, and the opposite effect of the MLS (for MLS and DiagMLS) is enhanced. In the 6X beam, an insufficient decrease of the mean energy is similarly present up to about 10 cm off-axis (the amount of lateral scatter was 7 cm). Moreover, the mean radial energy value on the central axis for the 6X is considerably lower for PddMLS and MLS. This indicates that the central axis mean energy value (at the level of the bottom of the flattening filter) is mostly determined by the large field PDDs.

The last of the primary source parameters, the intensity profile shape, also presents differences in the configuration for MLS conditions (Fig. 1), where higher energy fluences are shown with increasing off-axis distances.

#### Second source

The second source parameters are only available for the 6X configurations (the second source modeling is disabled for the FFF beams) and are reported in Table 1. The parameters are different in the various configurations. For example, the PinPoint presented the smallest source size and lowest intensity (for the acquisitions with SSD = 90 cm), while DiodeP configuration gave the largest source and highest intensity.

#### Electron contamination

**Fig. 2:**
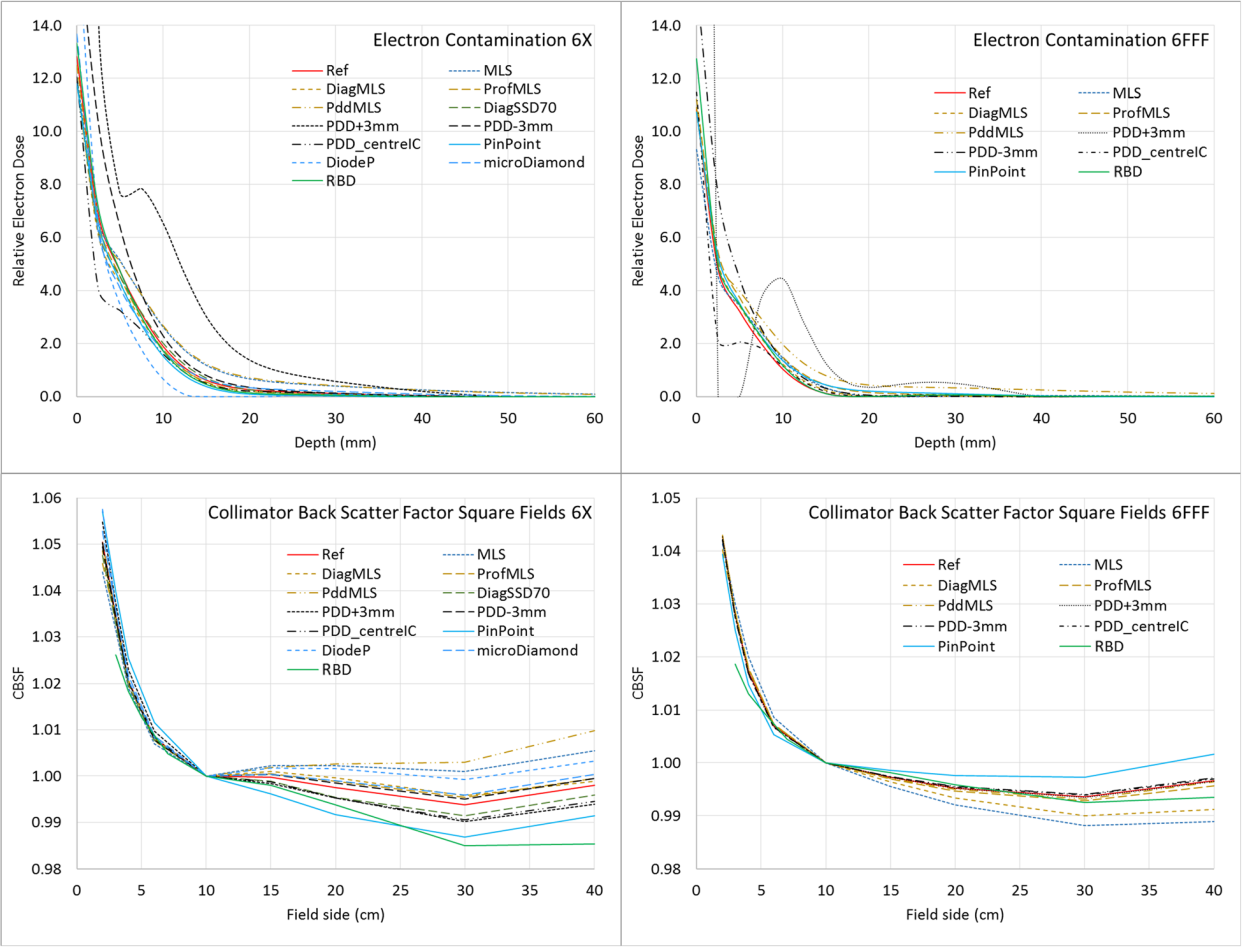
Electron contamination curves and collimator back scatter factors of square fields for the 6X (left column) and 6FFF beams (right column) in the different algorithm configurations

In Fig. 2 the depth-dependent electron contamination component is reported and in Table 1 the related parameters of σ_0_, σ_1_, and the fraction of σ_0_ relative to σ_1_. The vendor indications require that the curve should peak at a maximum depth of 0.5 mm, and should fall close to zero within 50 mm. The buildup region of the measured PDDs is used to model the electron contamination component. Consistently, the configurations with the incorrect zero settings in the vertical (depth) direction gave unacceptable results. The PDD + 3 mm (d_max_ shifted from 14 to 17 mm) showed a non-smoothly descending curve, a very high relative electron dose at the surface (94.0 and 113.5 – out of the plot scale – to compare with 12.8 and 10.8 of the Ref setting for the 6X and 6FFF, respectively); for the 6FFF the electron contamination falls to zero for depth bins at 2.5 and 5 mm. The PDD_centreIC curves show a discontinuity at 2.5 mm depth, that could be ascribed to the σ_0_, whose value is 350 and 81 in the PDD + 3 mm and PDD_centreIC cases, while it is in a range between 1 and 15 in all the other configurations (Fig. 2).

The electron contamination curve of the 6FFF beam seems to be more sensitive to the buildup measurements compared to 6X. This curve includes the dose in the buildup region that is not accounted for by the primary and secondary source components; since the second source is disabled in the FFF mode, the electron contamination also includes the low energy head scattering component, explaining these differences.

All the other configurations give similar electron contamination curves, with two differences: the MLS present higher curves, and the DiodeP gives a lower curve.

#### Collimator backscatter factor

The scanning curves are in principle not responsible for the CBSF table calculations since those factors are obtained from the measured output factors, and in the current work, the same measured output factors were used for all the configurations. However, some differences in the CBSF table were observed for the different configurations, derived from the different configuration parameters generated by the different situations, as shown in Fig. 2.

### Dose calculation accuracy

#### Calculations vs measurements used for configuration

The ability of the configuration process to accurately optimize the parameters was evaluated by comparing the calculated against the measured data used as input in the configuration process.

**Fig. 3:**
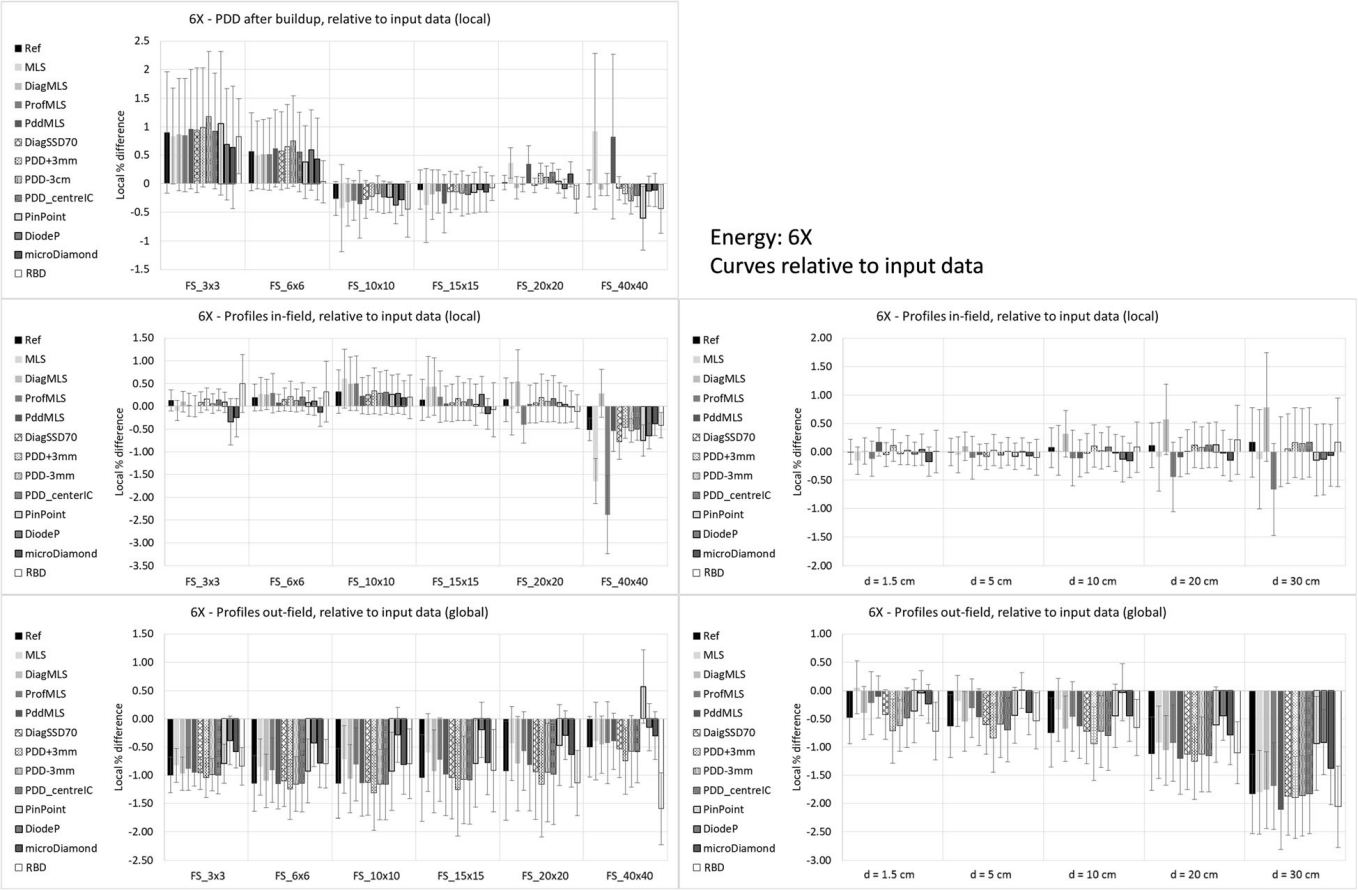
Differences calculated – measured input data for the 6X beam

**Fig. 4:**
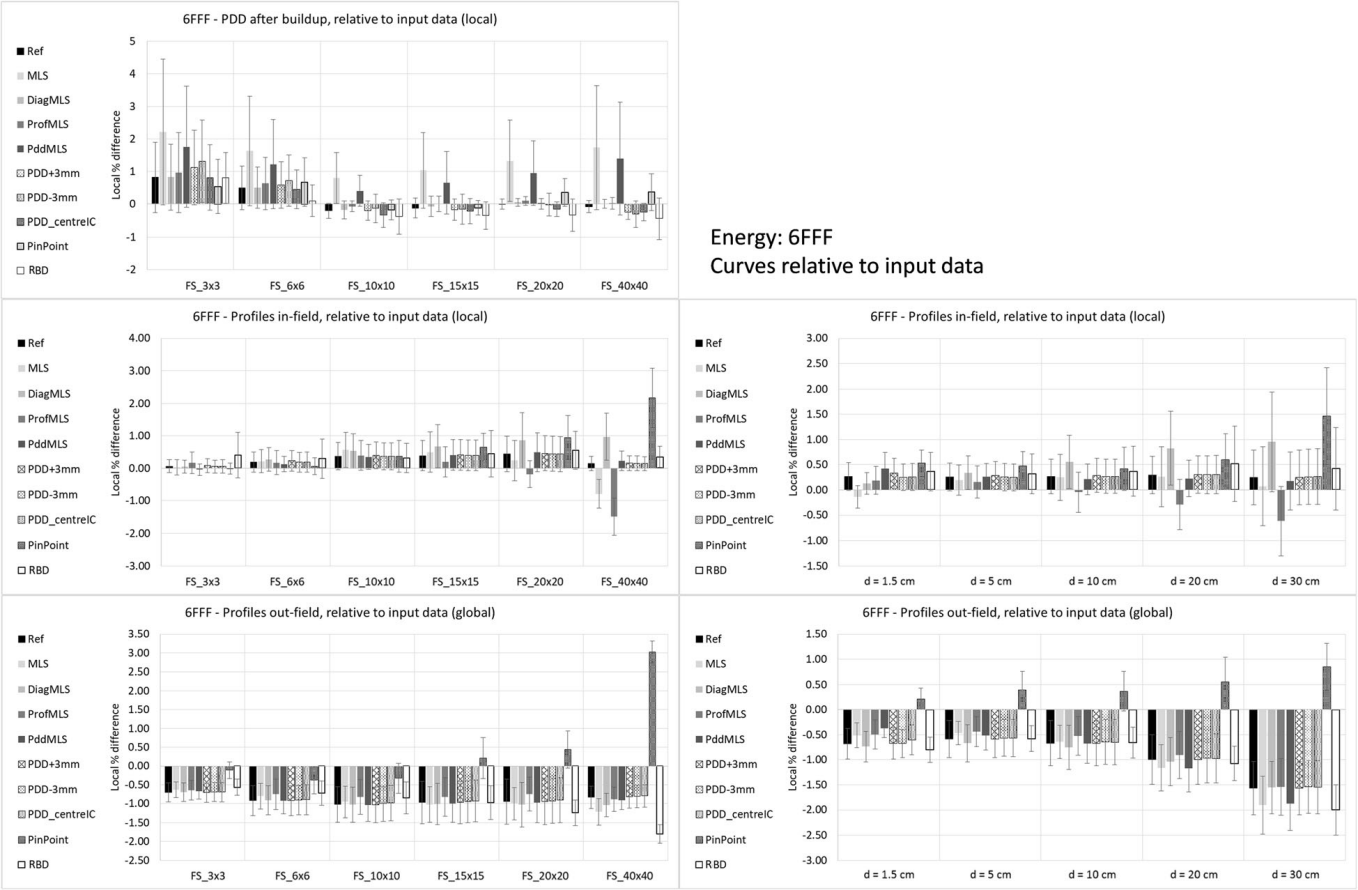
Differences calculated – measured input data for the 6FFF beam

Figures 3 and 4 report the mean differences for the two beams, 6X and 6FFF, respectively.

PDD differences are local (relative to the measured data) and refer to PDD renormalized to 100% at 10 cm depth, more reflecting the clinical situation of the dose calculation. The mean values and standard deviations are computed for each field size and for each configuration. The average agreement is rather constant for medium-large fields, while there is a trend to overestimate the dose for smaller fields for most of the configurations. The MLS (and Pdd_MLS) overestimates the dose more than the other configurations for all field sizes for 6FFF, and for the largest field for 6X.

Profiles were analyzed in two regions, in-field and out-of-field. The in-field differences were local (relative to the measured data), while the out-field were global (relative to the central axis dose), to reflect the clinical importance. The plots on the left side in the Figures show data stratified per field size, and the mean difference is the mean value over all the depths and over all the points in the region of interest. The reported standard deviation is the mean of the standard deviations for each profile. On the right side of the Figures, the stratification is by depth, averaged over all the field sizes. Inside the field, the MLS (and ProfMLS) presented an underestimation of the mean dose along the profile; the PinPoint for the 6FFF beam had the largest difference for the large field and large depth. All the other configurations were similarly able to reproduce the measured data. Outside the beam, the calculations underestimated the dose in all cases, except the PinPoint for the 6FFF, especially for the large field, and increasing with depth.

Table 2 reports the summary of the mean, standard deviation, and range of the average differences with respect to input data. It quantifies what is shown in the plots: the global mean for the PDDs was within 0.4% in all the cases except the MLS (and PddMLS for the 6FFF only), where the values were larger than 1%.

**Table 2:** Mean differences between calculated and input measured data, as percentage relative to the measured point for the PDD after the buildup region (d > 18 mm) and the profiles inside the field (5 mm per side inside the nominal field side rescaled according to the depth). Data represents the average (of the means over all the test points of each curve) on all the conditions of field size and depth, ± the standard deviation, and the range

	6X		6FFF	
	PDD vs. input	Profiles vs. input	PDD vs. input	Profiles vs. input
Ref	0.18 ± 0.41 [−0.26,0.90]	0.07 ± 0.32 [− 0.57, 0.76]	0.15 ± 0.37 [− 0.21,0.82]	0.27 ± 0.19 [− 0.16,0.79]
MLS	0.30 ± 0.53 [− 0.43,0.92]	− 0.08 ± 0.85 [−2.93, 1.31]	1.46 ± 0.47 [0.80,2.21]	0.13 ± 0.54 [−1.65,0.94]
DiagMLS	0.11 ± 0.43 [− 0.32,0.86]	0.36 ± 0.42 [− 0.43,1.39]	0.19 ± 0.36 [− 0.18,0.83]	0.56 ± 0.52 [− 0.12,1.94]
ProfMLS	0.15 ± 0.40 [− 0.30,0.85]	− 0.29 ± 1.14 [−4.53, 0.91]	0.28 ± 0.38 [− 0.07,0.96]	−0.12 ± 0.87 [−3.68,0.61]
PddMLS	0.34 ± 0.53 [−0.36,0.96]	− 0.02 ± 0.35 [− 0.96, 0.62]	1.07 ± 0.45 [0.41,1.76]	0.26 ± 0.25 [− 0.22,0.79]
DiagSSD70	0.16 ± 0.44 [− 0.28,0.94]	−0.02 ± 0.39 [− 0.96, 0.69]		
PDD + 3 mm	0.21 ± 0.46 [− 0.22,0.99]	0.11 ± 0.30 [−0.56, 0.71]	0.18 ± 0.51 [− 0.25,1.13]	0.29 ± 0.20 [− 0.18,0.78]
PDD-3 mm	0.23 ± 0.55 [− 0.30 ± 1.17]	0.03 ± 0.32 [− 0.62, 0.74]	0.24 ± 0.59 [− 0.31,1.32]	0.27 ± 0.20 [− 0.15,0.81]
PDD_centerIC	0.17 ± 0.44 [− 0.24,0.92]	0.08 ± 0.32 [− 0.56, 0.74]	0.05 ± 0.43 [− 0.34,0.81]	0.27 ± 0.19 [− 0.13,0.82]
PinPoint	0.08 ± 0.53 [− 0.61,1.06]	− 0.03 ± 0.43 [− 1.66, 0.65]	0.27 ± 0.32 [− 0.18,0.68]	0.70 ± 0.96 [− 0.40,3.96]
DiodeP	0.10 ± 0.40 [− 0.38,0.69]	− 0.05 ± 0.42 [− 1.23, 0.80]		
microDiamond	0.11 ± 0.33 [− 0.29,0.64]	− 0.12 ± 0.23 [− 0.53,0.39]		
RBD	−0.06 ± 0.44 [− 0.45,0.83]	0.07 ± 0.36 [− 0.54,0.69]	− 0.10 ± 0.44 [− 0.45,0.80]	0.40 ± 0.19 [− 0.01,0.83]

Regarding the profiles, the ranges present differences larger than 2% for 6X in the ProfMLS (− 4.5%), MLS (− 2.9%), and for the 6FFF in the ProfMLS (− 3.7%) and PinPoint (+ 4.0%) configurations. The configuration process, especially in the MLS conditions, is unable to adjust the parameters to reproduce a similar beam.

Relative to the input data, the configurations with all average values < 1% were: Ref, PDD_centerIC, RBD, DiagSSD70 and microDiamond (only 6X for the last two).

#### Calculations versus reference measurements

More interesting from the clinical viewpoint is the comparison between calculations and the reference measured data, that we assume as the actual beam.

**Fig. 5:**
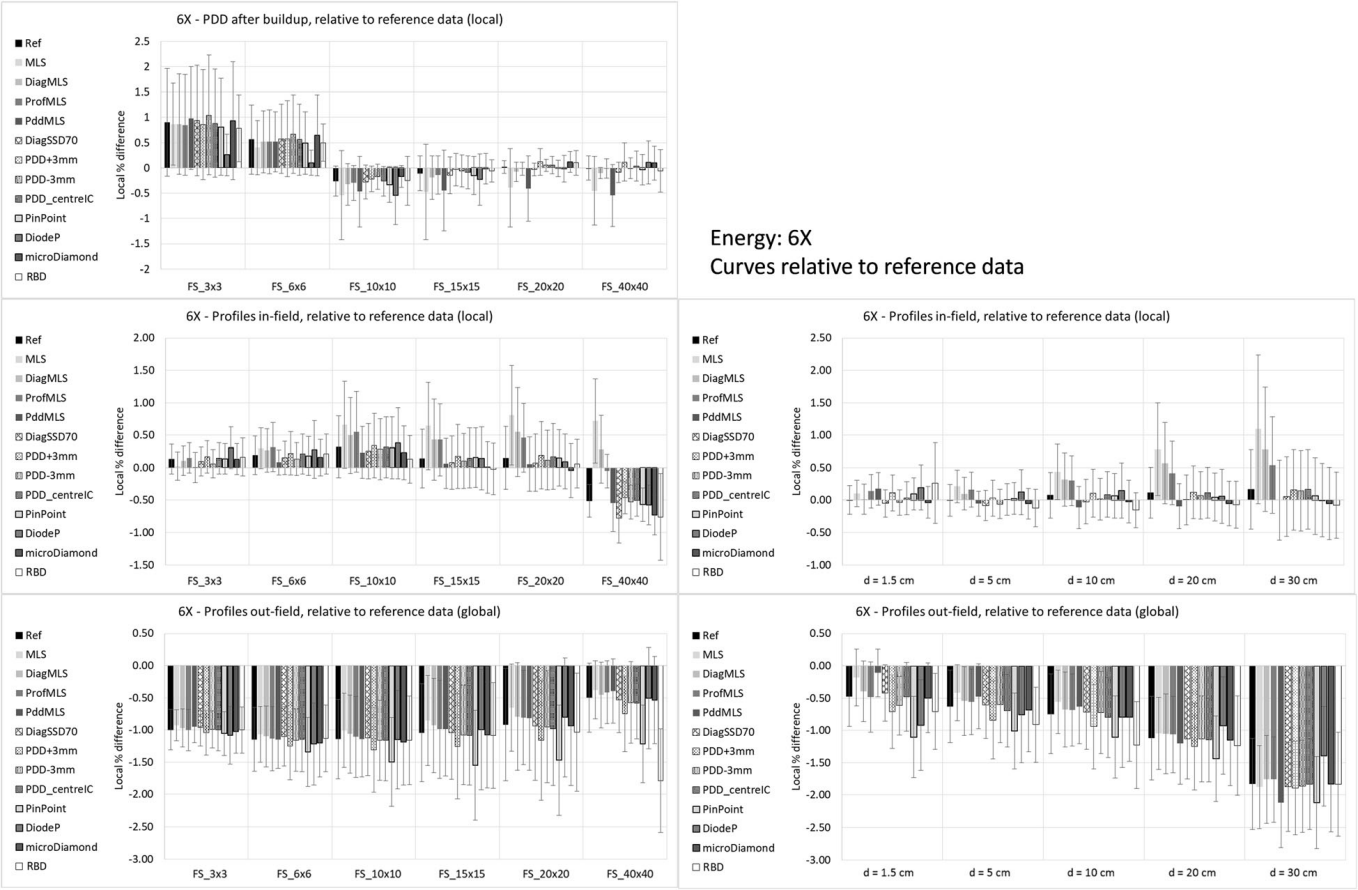
Differences calculated – measured reference data for the 6X beam

**Fig. 6:**
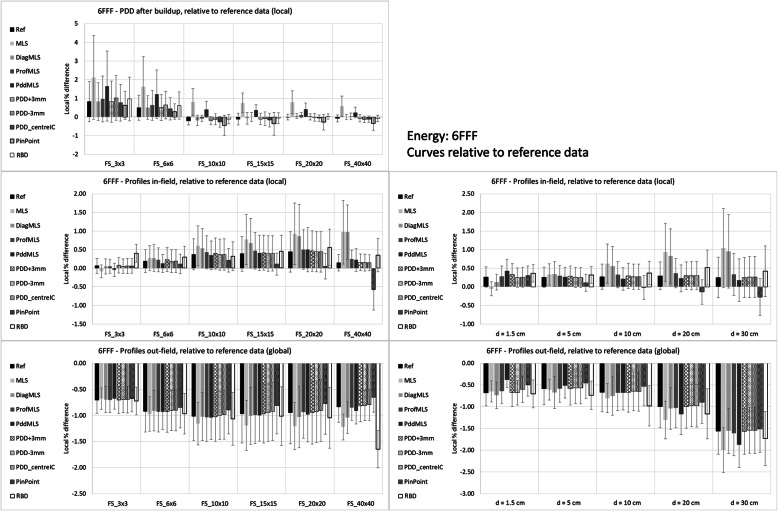
Differences calculated – measured reference data for the 6FFF beam

Figures 5 and 6 show the differences as in Figs. 3 and 4 for the 6X and 6FFF, respectively, but compared with the Ref measurement set (RBD curves were recomputed at SSD = 90 cm). The configurations presenting larger discrepancies were those in MLS conditions and, to some extent, the PinPoint-3D for the 6FFF.

Table 3 summarized the data like Table 2, related to the reference measurements. The mean value for the PDDs > 1% was reported only for the MLS configuration for 6FFF, with also a profile difference > 2%.

**Table 3:** Mean differences between caculated and reference measured data, as percentage relative to the measured point for the PDD after the buildup region (d > 18 mm) and the profiles inside the field (5 mm per side inside the nominal field side rescaled according to the depth). Data represents the average on all the conditions of field size and depth. Data represents the average (of the means over all the test points of each curve) on all the conditions of field size and depth, ± the standard deviation, and the range

	6X		6FFF	
	PDD vs. reference	Profiles vs. reference	PDD vs. reference	Profiles vs. reference
Ref	0.18 ± 0.41 [−0.26,0.90]	0.07 ± 0.32 [− 0.57,0.76]	0.15 ± 0.37 [− 0.21,0.82]	0.27 ± 0.19 [− 0.16,0.79]
MLS	− 0.10 ± 0.54 [− 0.54,0.86]	0.53 ± 0.56 [− 0.18,1.85]	1.11 ± 0.56 [0.57,2.11]	0.58 ± 0.65 [− 0.64,2.12]
DiagMLS	0.11 ± 0.43 [− 0.32,0.86]	0.36 ± 0.42 [− 0.43,1.39]	0.19 ± 0.36 [− 0.18,0.83]	0.56 ± 0.52 [− 0.12,1.94]
ProfMLS	0.15 ± 0.40 [− 0.30,0.85]	0.31 ± 0.32 [− 0.29,1.20]	0.28 ± 0.38 [− 0.07,0.96]	0.32 ± 0.21 [− 0.07,0.91]
PddMLS	−0.06 ± 0.59 [− 0.55,0.98]	−0.02 ± 0.35 [− 0.96,0.62]	0.72 ± 0.53 [0.24,1.66]	0.26 ± 0.25 [− 0.22,0.79]
DiagSSD70	0.16 ± 0.44 [− 0.28,0.94]	−0.02 ± 0.39 [− 0.96,0.69]		
PDD + 3 mm	0.24 ± 0.37 [−0.22,0.86]	0.11 ± 0.30 [−0.56,0.71]	0.16 ± 0.37 [− 0.20,0.83]	0.29 ± 0.20 [− 0.18,0.78]
PDD-3 mm	0.26 ± 0.45 [− 0.17,1.05]	0.03 ± 0.32 [− 0.62,0.74]	0.22 ± 0.45 [− 0.13,1.02]	0.27 ± 0.20 [− 0.15,0.81]
PDD_centerIC	0.20 ± 0.40 [− 0.26,0.88]	0.08 ± 0.32 [− 0.56,0.74]	0.10 ± 0.37 [− 0.26,0.76]	0.27 ± 0.19 [− 0.13,0.82]
PinPoint	0.13 ± 0.39 [− 0.33,0.81]	0.06 ± 0.33 [− 0.87,0.69]	−0.09 ± 0.40 [− 0.44,0.62]	−0.01 ± 0.41 [− 1.52,0.49]
DiodeP	− 0.05 ± 0.27 [− 0.55,0.26]	0.10 ± 0.35 [− 0.90,0.52]		
microDiamond	0.27 ± 0.39 [− 0.17,0.94]	− 0.04 ± 0.35 [− 0.98,0.50]		
RBD	0.17 ± 0.36 [− 0.25,0.79]	− 0.04 ± 0.44 [− 1.14,0.63]	0.22 ± 0.41 [− 0.12,0.97]	0.06 ± 0.33 [− 1.23,0.60]

**Fig. 7:**
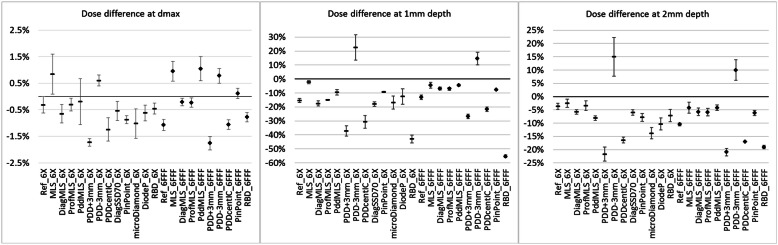
Differences calculated – measured reference data at d_max_, 1 and 2 mm depth

Regarding the dose close to the surface and at d_max_, Fig. 7 shows the dose difference, relative to the reference, at the d_max_ (PPDs normalized to 10 cm depth), and at 1 and 2 mm depth.

In general, the configurations with intentional acquisition errors had inaccuracies relative to the input dataset larger than in the comparison against the reference dataset.

## Discussion

Some of the most common and possible errors in the scanning measurements were deliberately made here to acquire datasets for beam configuration, in order to evaluate their impact on the Acuros dose calculation algorithm configuration, relative to an assumed correct (Ref) dataset.

The missing lateral scatter is the most common possible error since the available water phantoms are not large enough to entirely accommodate diagonal profiles (for a 40 × 40 cm^2^ field, collimator to 45°, at 30 cm depth would require a 75–80 cm scan for SSD = 90 cm, 80–85 cm scan for SSD = 100 cm). A classical solution adopted for these scans is to position the phantom off-center, with the central axis closer to the phantom wall; this solution prevents the complete production of the phantom lateral scatter. The acquisition of the diagonal profiles along the beam diagonal (without collimator rotation) could lead to data not fully compatible with the non-diagonal profiles: only the acquisition across the same line can guarantee the same conditions of symmetry. This enforces the rationale for acquiring the diagonal profiles with the 45° collimator rotation, and a setup solution is needed. The MLS conditions yielded low-quality configurations, with systematic errors > 2% in some conditions, suggesting that high attention has to be paid to the lateral scatter conditions. A solution different from the off-axis phantom position is the acquisition of the large profiles at a shorter SSD to reduce the field size, and then geometrically rescale the profiles, as done in the DiagSSD70 configuration. This setting presents conditions which can change the head scatter, and the Ref and the DiagSSD70 configurations are indeed not identical, as well as the acquired diagonals (Supplementary Material). However, this solution resulted in an acceptable configuration. This demonstrate that it is possible to acquire the diagonal profiles at a shorter distance with no risk of missing lateral scatter.

Regarding the effective point of measurement for PDDs, the incorrect setting of the EPoM leads to an unacceptable electron contamination modeling and consequently to an incorrect estimation of the dose in the buildup region. However, this error has consequences also at larger depths, as shown in Tables 2 and 3.

Another important point is the proper choice of the detector for data acquisition. Small field measurements are outside the scope of this work. In principle, ion chambers are the gold standard for any photon dosimetry. Of course, large ion chambers suffer from the volume effect, and smaller chambers are to be preferred. This effect overestimates the beam penumbra; however, the calculated penumbra is not determined by the measurements. It can be adjusted in the beam configuration of the algorithm by tuning two parameters, called Effective spot size, X and Y: the larger the spot, the larger the penumbra. For the current work, we used the parameters suggested by the vendor (1 mm for both directions for jaw defined fields in Acuros [23]). Adjusting these parameters, which have effects also on the MU calculations for small fields, was outside the scope of the present study. This subject has been studied in different works [17, 18, 21, 24–26]. However, the small sensitive volume could make those ion chambers more susceptible to a polarity effect, especially for small fields, or outside the primary beam. The polarity effect of the ion chambers used in this work, according to the vendor specifications, is less than 0.8% (and also proven with measurements for this work) [27, 28].

Also, the possible detector energy dependence should be evaluated, since it could lead to inaccuracies outside the primary beam and in the primary beam at large depths for large fields; this could be the case for some diodes.

Finally, the RBD could be considered an interesting solution for the beam configuration. The comparison with respect to the other configurations based on the clinical beam measurements was similar to the input data. However, a careful check of calculated against measured data in clinical conditions is recommended. In this work, the profiles presented differences larger than 1% in some conditions, which was not the case for the other sets based on measurements. This might be ascribed to the non-clinical SSD (100 cm) of the data acquired for the available dataset.

However, it is outside the scope of this work to give suggestions on what should absolutely be measured if using the vendor-supplied data. For this, we suggest following the recommendations on quality assurance of the treatment planning systems (for example by the IAEA or the AAPM).

This work has a number of known limitations. First of all, it refers to broad beams, no small field was analyzed, but the aim of the study was on the general, non-small fields, that, on the contrary, require special attention. Secondly, data and calculations do not include fields shaped by the MLC: the whole configuration of the algorithms in Eclipse is done by non-MLC fields, and the specific MLC-related parameters and handling have been the subject of other publications [18, 29, 30]. Then, only 6MV beams were analyzed to limit the already huge amount of data to present. The last point to mention is the use of the same output factor acquired data for all the configurations. The monitor units are mainly affected by different output factor measurements, and the goal of this work was to evaluate relative dose only. The effect of different output factor data has been the subject of other works, mostly in the frame of small field management [14, 18, 29].

Finally, the choice of Ref dataset as a reference does not mean that it is a perfect representation of the beam. The work intended to evaluate the different configurations with respect to a reasonable set of measurements, and all the results can be seen in a relative way.

No clinical tests have been presented in this work. It is of course interesting to know if the difference between the model parameters obtained in the beam modeling is reflected in the clinical plans. This next step is currently under investigation within our group.

A short summary of the most important effect of the different beam data on the configuration model can be delineated:

- *PDDs*: if they are acquired in incomplete lateral scatter conditions, it can result in an erroneous penetration for large fields; if acquired in incorrect EPoM, it can result in erroneous build-up dose estimation;

- *profiles*: the second scatter source is modeled by the profiles. Attention has to be paid to the out-of-field measurements, using an appropriate detector;

- *diagonals*: they are used to estimate the beam quality. An incomplete lateral scatter can produce inaccurate beam modeling.

## Conclusion

A number of configurations for the Acuros dose calculation algorithm were optimized for a 6 MV beam in both flattened and FFF mode, using dataset acquired with intentional errors or different detectors. The conditions of missing lateral scatter and incorrect setting of the effective point of measurement could lead to erroneous configurations and should be avoided. The choice of the detector has to be appropriate. Whenever the representative beam data has to be used, a careful check under more clinical geometrical conditions is necessary.

## Supplementary information

**Additional file 1.**

## Data Availability

The datasets supporting the conclusions of this article are included within the article and its additional file. Additional information are available on request.

## Abbreviations

FFF: Flattening filter free
MLS: Missing lateral scatter
EPoM: Effective point of measurement
TCP: Tumour control probability
NTCP: Normal tissue complication probability
PDD: Percentage depth dose
MLC: Multileaf collimator
d_max_: Depth of maximum dose
SSD: Source to surface distance
AAA: Anisotropic analytical algorithm
MU: Monitor unit
CBSF: Collimator backscatter factor
Ref: Reference
RBD: Representative beam data
