# Supplementary material for: The impact of scanning data measurements on the Acuros dose calculation algorithm configuration
Source: Radiat Oncol. 2020 Jul 10;15:169. doi: 10.1186/s13014-020-01610-7 (PMC7350585; doi:10.1186/s13014-020-01610-7)

The impact of scanning data measurements on the Acuros dose calculation algorithm configuration

A. Fogliata, E. Esposito, L. Paganini, G. Reggiori, S. Tomatis, M. Scorsetti, L. Cozzi

Supplementary Material

In this supplementary material document, the data, in form of PDDs and profiles, are reported.
In the first part, from Figure 1S to Figure 5S, the measured data are presented, acquired using different detectors or different setup conditions (the missing lateral scatter, MLS, with only 7 cm of phantom on one side of the beam central axis).
In the second part, from Figure 6S to Figure 11S, the same PDDs and profiles are shown for the different configurations, as explained in the main text, together with the measured reference data, i.e. the Semiflex-3D data, that was assumed to better reflect the actual beam.

Figure 1S - PDDs: 6X measured, 3x3 and 40x40 cm^2 with different detectors, and in conditions of missing lateral scatter MLS, with the Semiflex3D. The first plot is normalized to d_{max}, the second at 10 cm depth ("clinical" conditions).

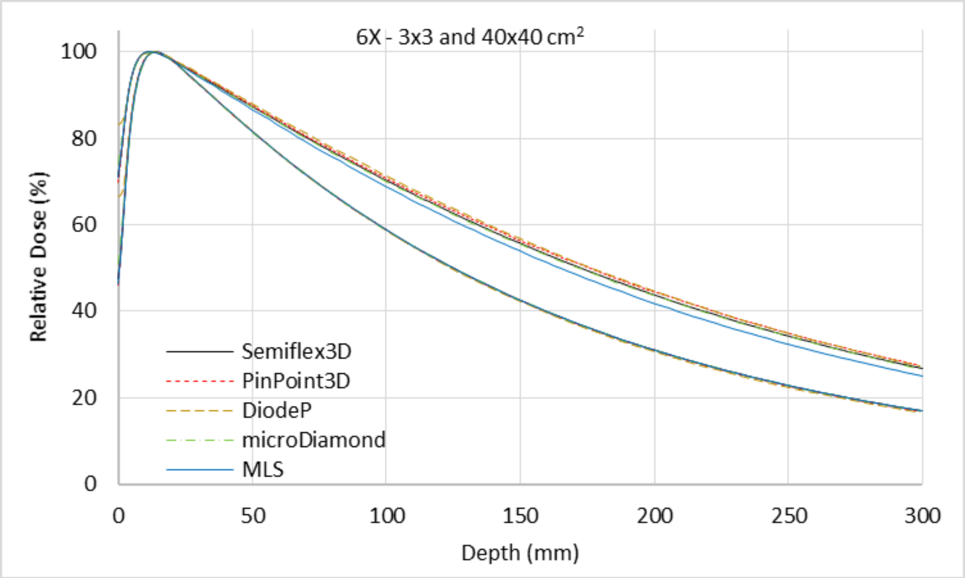

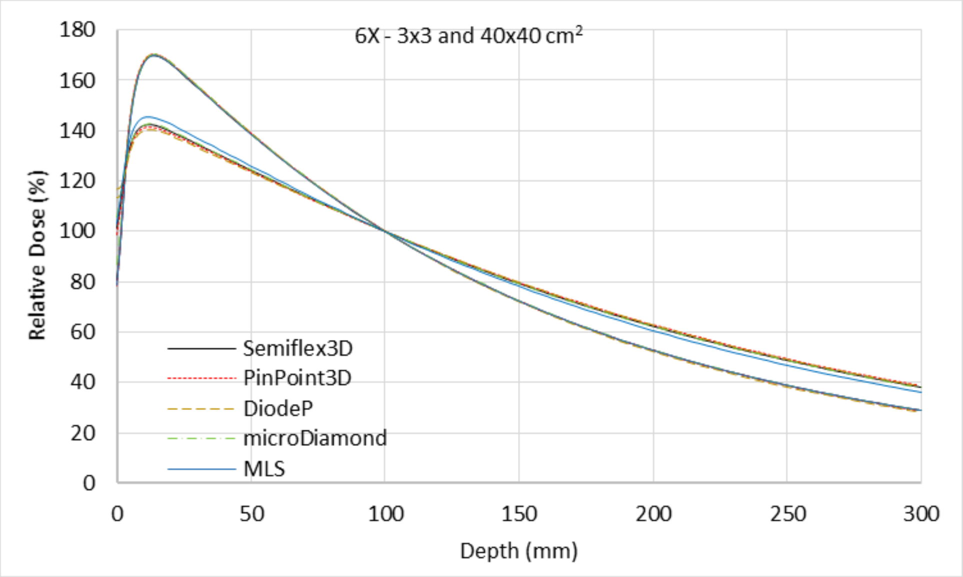

Figure 2S - PDDs: 6FFF measured, 3x3 and 40x40 cm^2 with different detectors, and in conditions of missing lateral scatter MLS, with the Semiflex3D. The first plot is normalized to d_{max}, the second at 10 cm depth ("clinical" conditions).

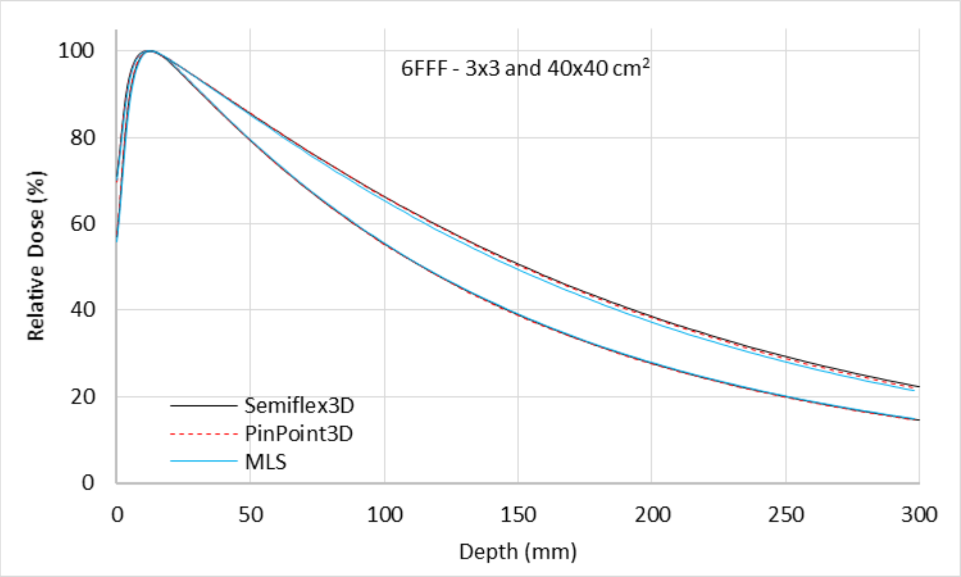

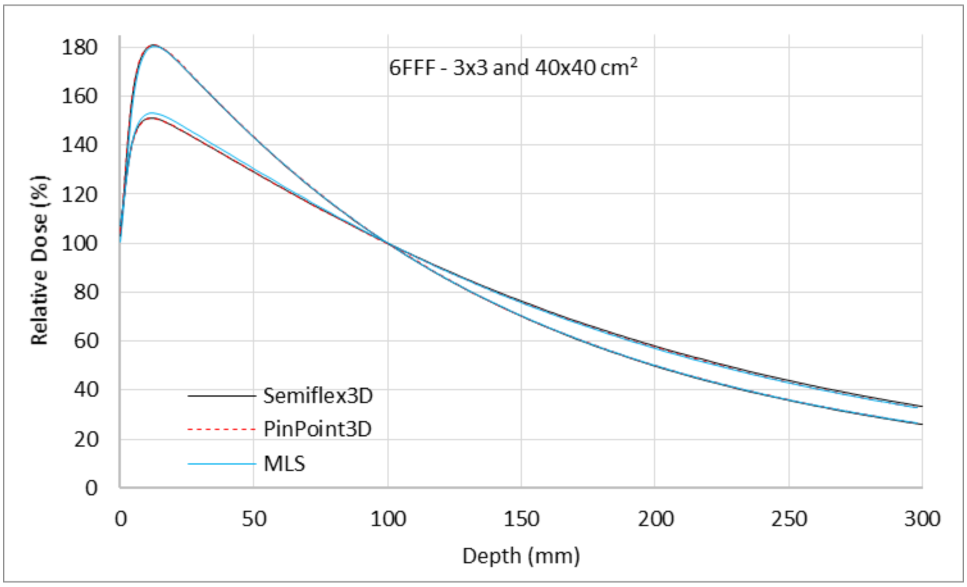

Figure 3S - Profiles: 6X measured, 10x10 and 40x40 cm^2 with different detectors, and in conditions of missing lateral scatter MLS with the Semiflex3D. First plot: d_{max}; second plot: 10 cm depth; third plot: 30 cm depth.

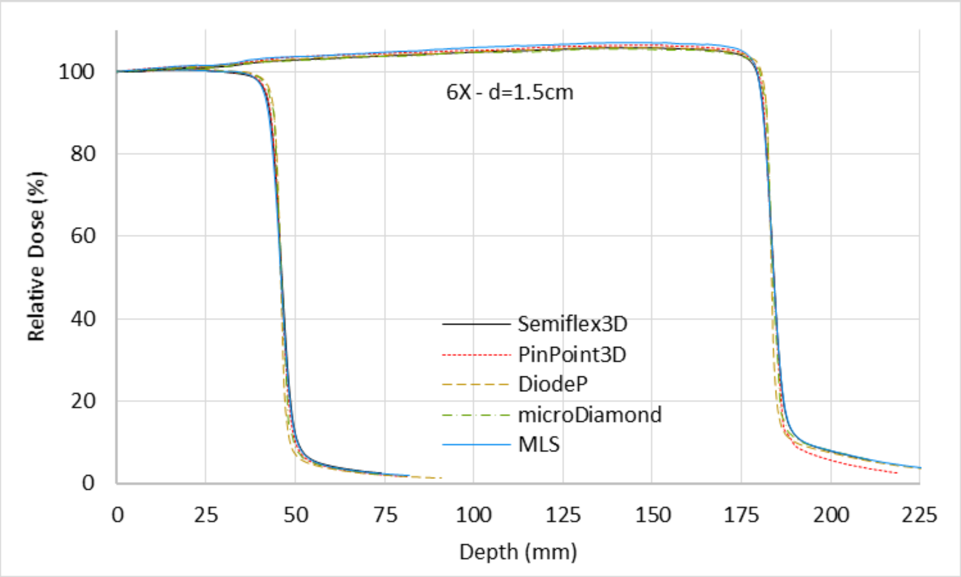

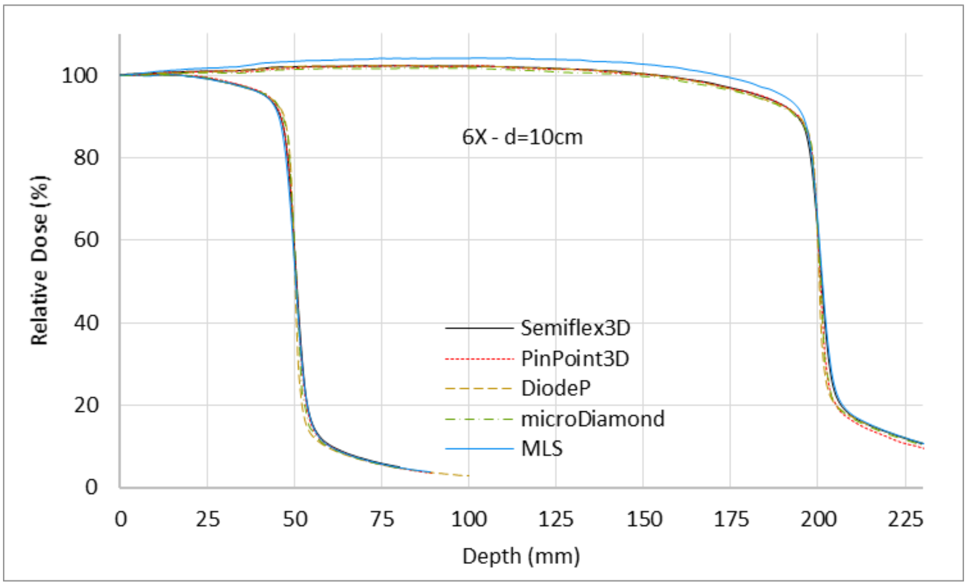

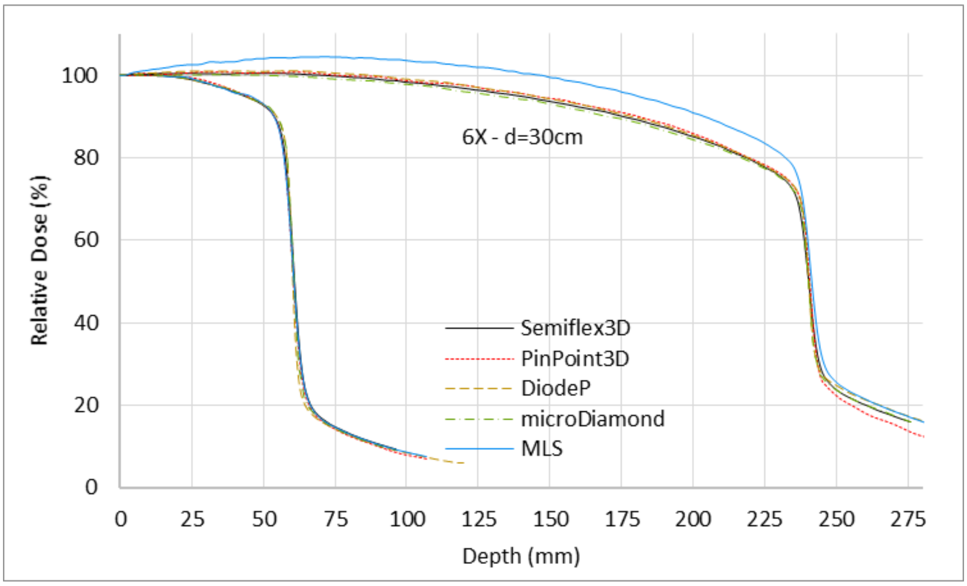

Figure 4S - Profiles: 6FFF measured, 10x10 and 40x40 cm^2 with different detectors, and in conditions of missing lateral scatter MLS with the Semiflex3D. First plot: d_{max}; second plot: 10 cm depth; third plot: 30 cm depth.

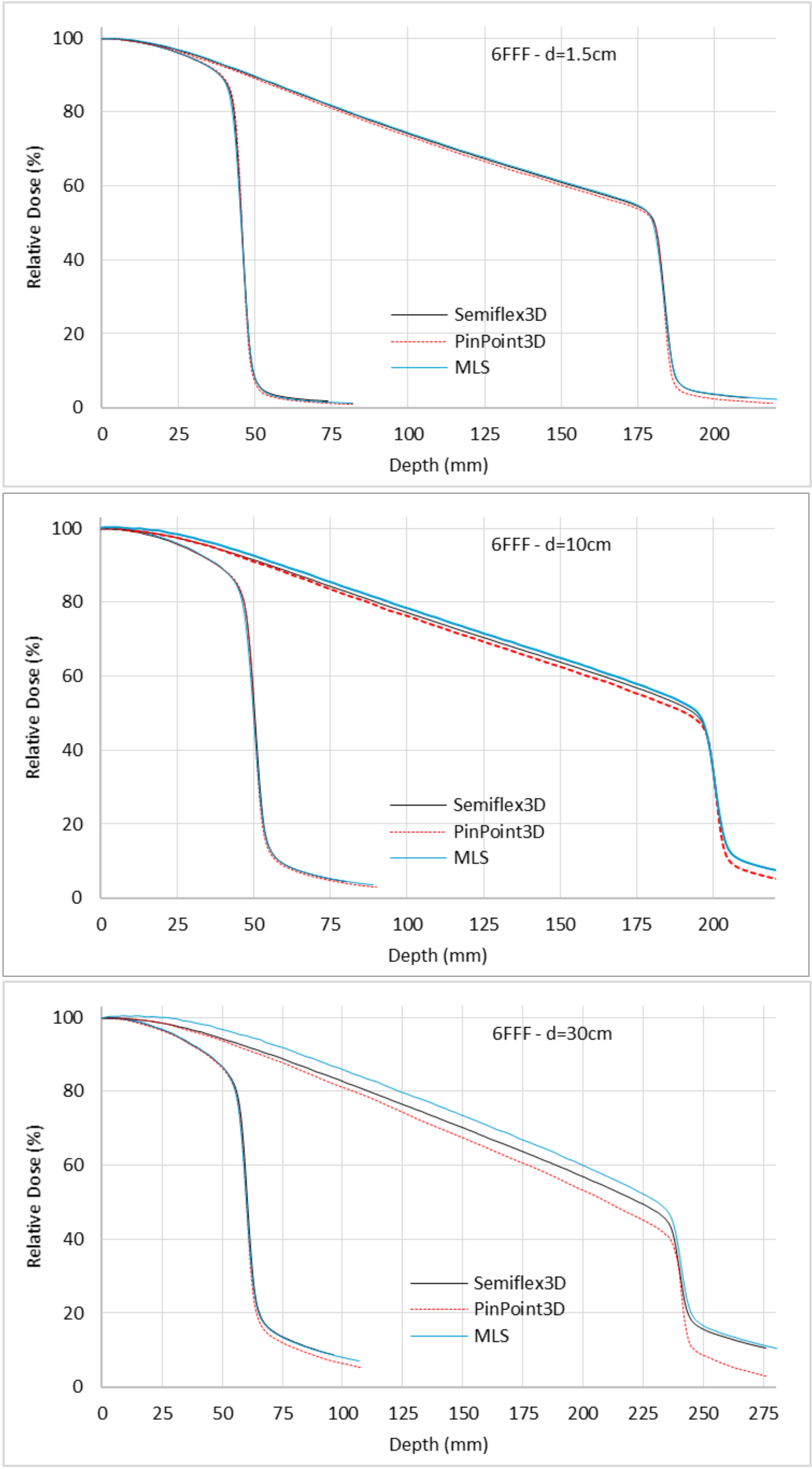

Figure 5S - Profiles: 6X measured diagonal profiles with the Semiflex3D, at SSD=90 cm and at SSD=70 cm geometrically rescaled to SSD=90 cm. First plot: d_{max}; second plot: 10 cm depth; third plot: 30 cm depth.

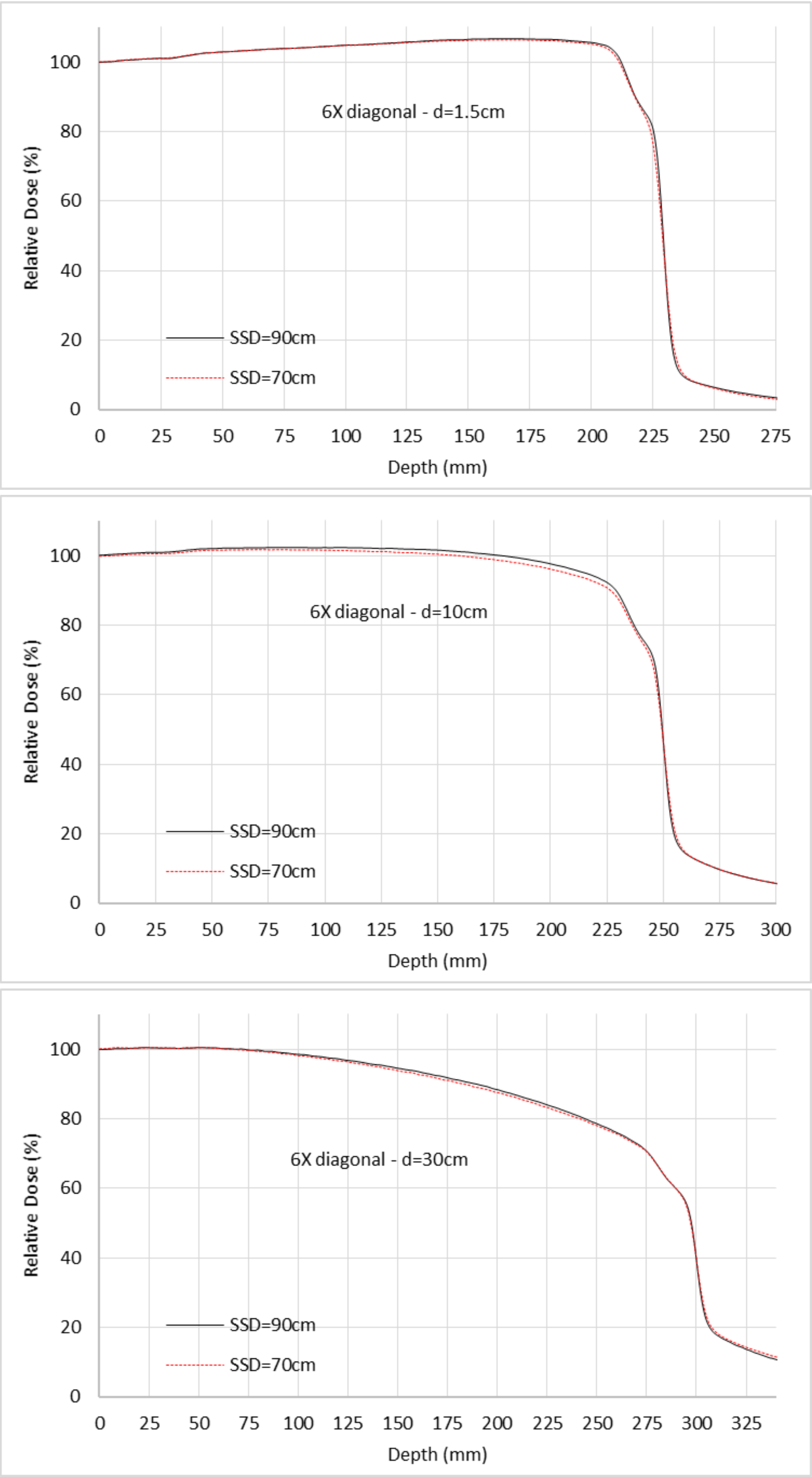

Figure 6S - PDDs: 6X, 3x3 cm^2 calculated by Acuros in the different configurations. “Measurements” refers to the reference Semiflex3D measured data for comparison. The first plot is normalized to d_{max}, the second at 10 cm depth (“clinical” conditions). For the configurations in missing lateral scatter conditions, only the MLS is here reported (no ProfMLS, DiagMLS, PddMLS) for sake of simplicity, here and in the following Figures.

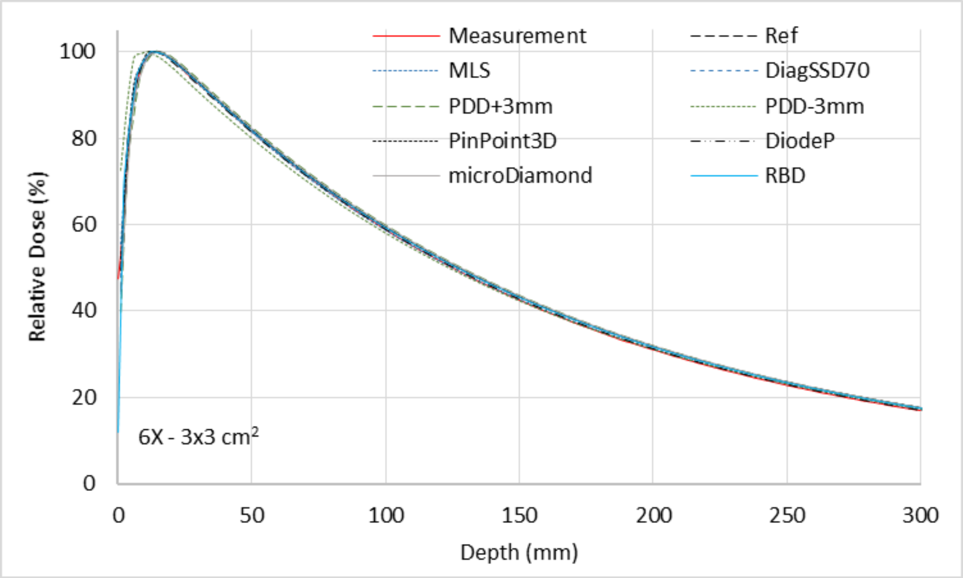

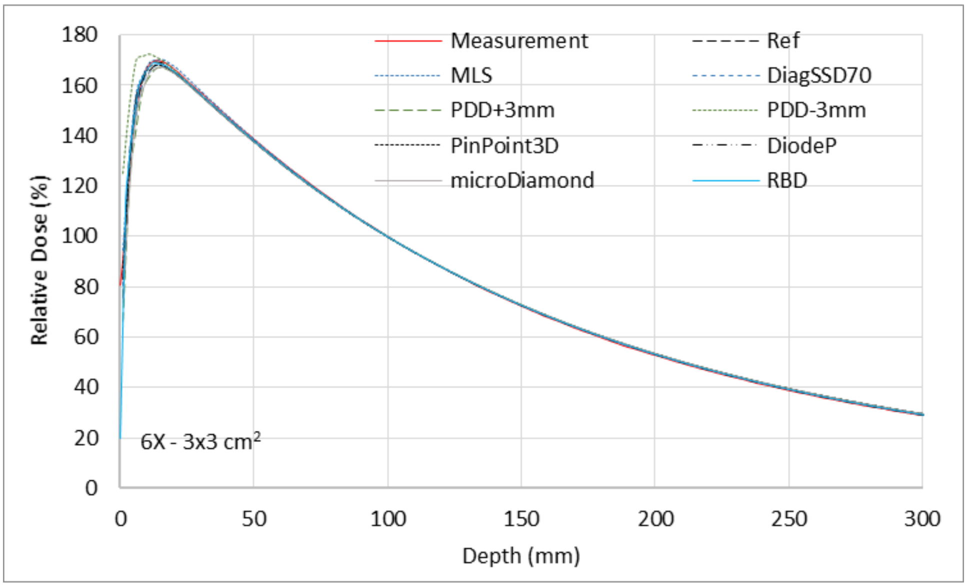

Figure 7S - PDDs: 6X, 40x40 cm^2 calculated by Acuros in the different configurations. "Measurements" refers to the reference Semiflex3D measured data for comparison. The first plot is normalized to d_{max}, the second at 10 cm depth ("clinical" conditions).

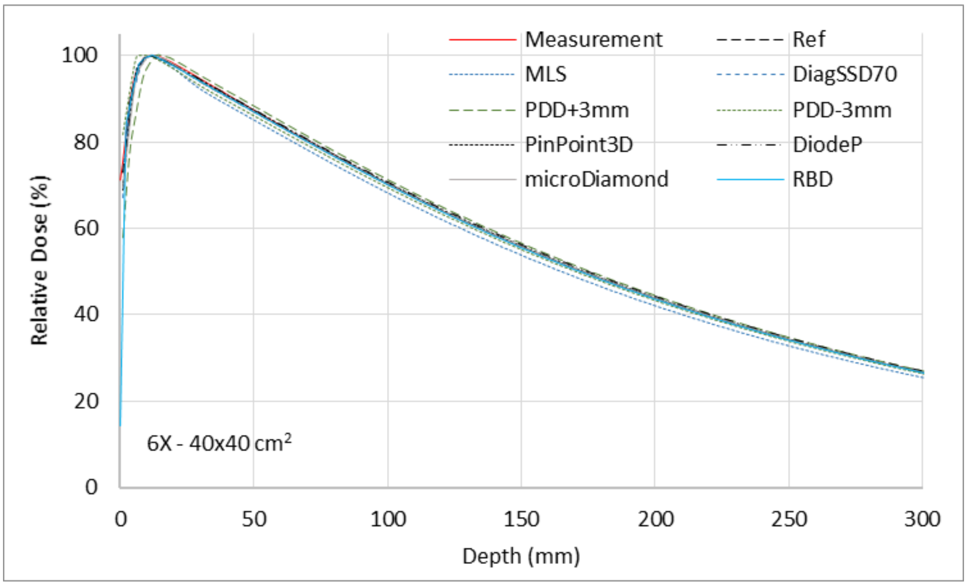

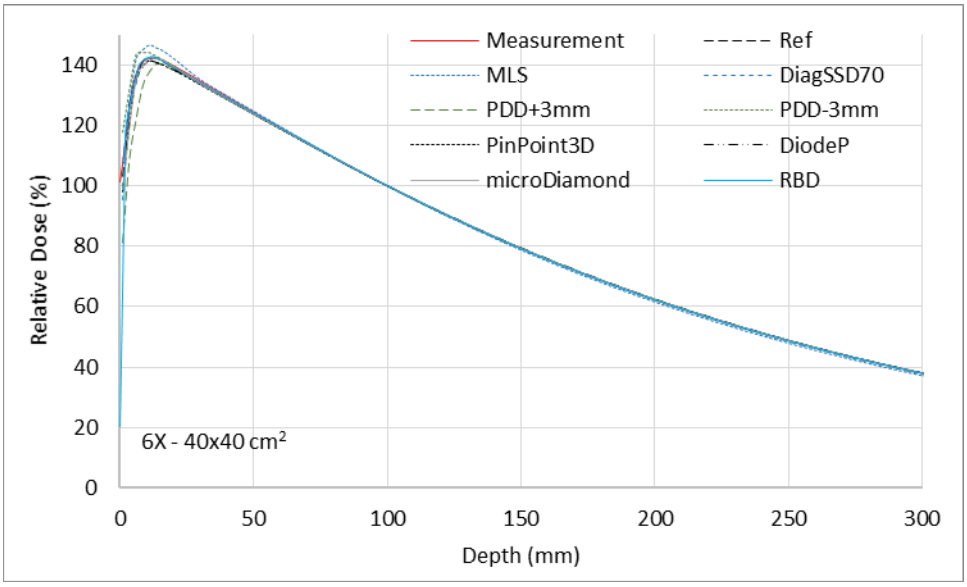

Figure 8S - PDDs: 6FFF, 3x3 cm^2 calculated by Acuros in the different configurations. “Measurements” refers to the reference Semiflex3D measured data for comparison. The first plot is normalized to d_{max}, the second at 10 cm depth (“clinical” conditions).

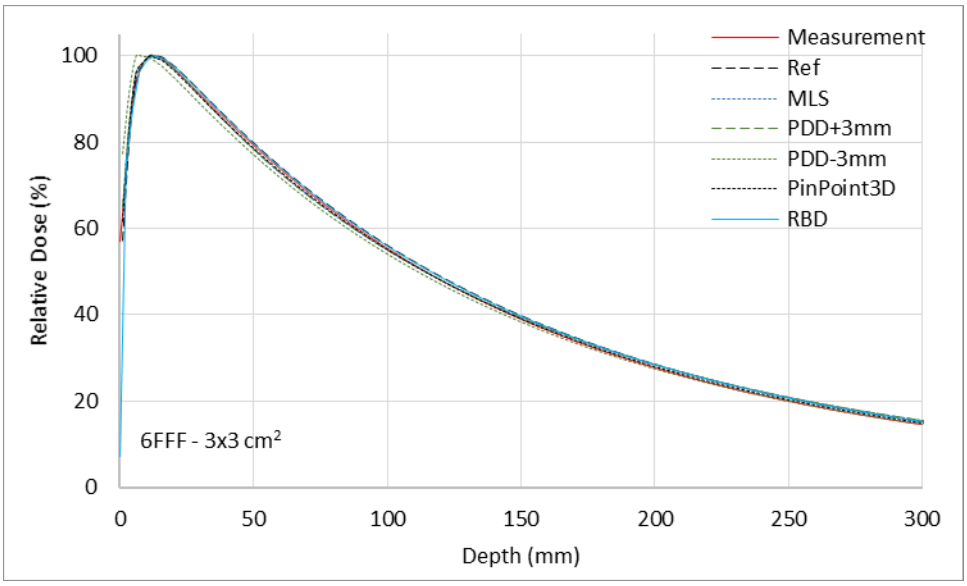

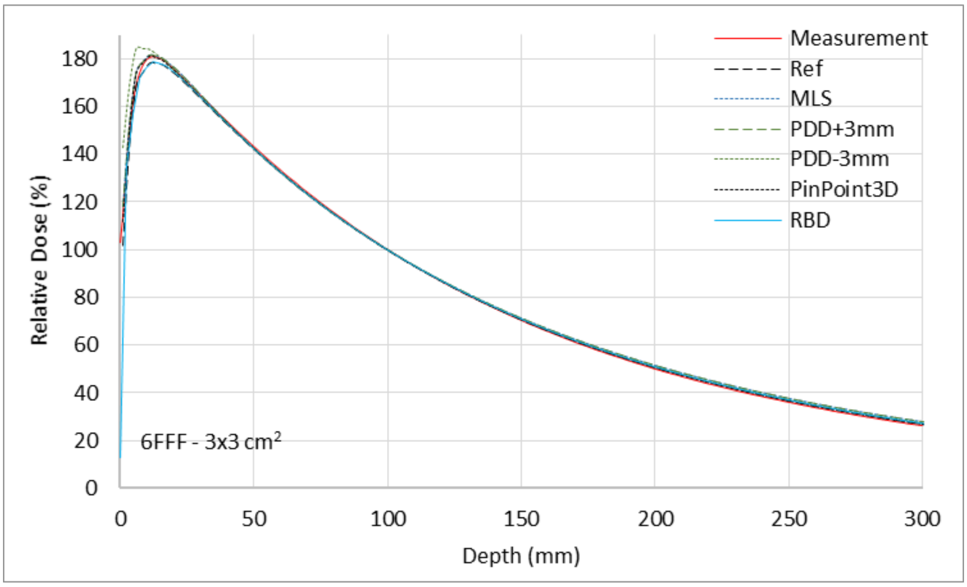

Figure 9S - PDDs: 6FFF, 40x40 cm^2 calculated by Acuros in the different configurations. “Measurements” refers to the reference Semiflex3D measured data for comparison. The first plot is normalized to d_{max}, the second at 10 cm depth (“clinical” conditions).

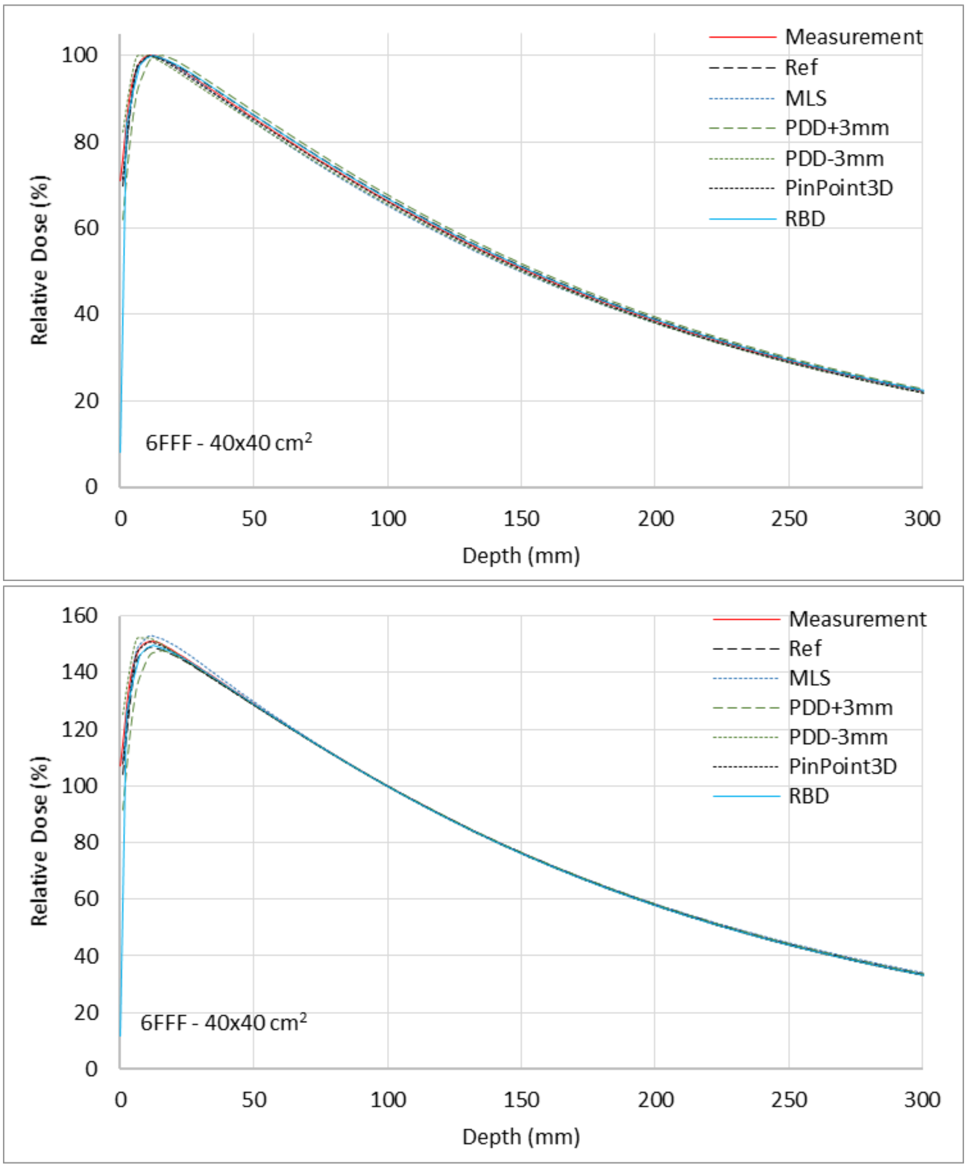

Figure 10S - Profiles: 6X, 10x10 and 40x40 cm^2 calculated by Acuros in the different configurations. "Measurements" refers to the reference Semiflex3D measured data for comparison. First plot: d_{max}; second plot: 10 cm depth; third plot: 30 cm depth.

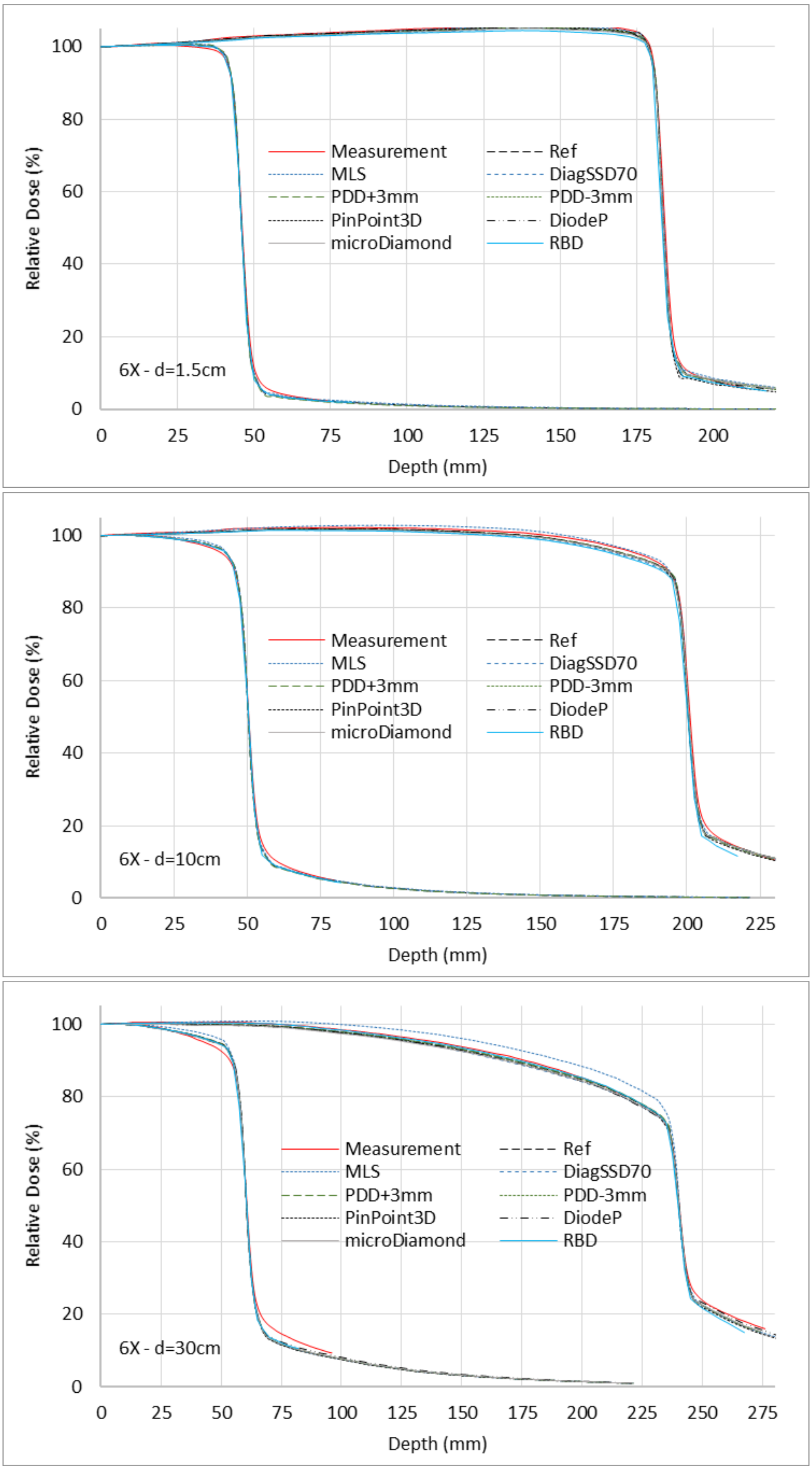

Figure 11S - Profiles: 6X, 10x10 and 40x40 cm^2 calculated by Acuros in the different configurations. "Measurements" refers to the reference Semiflex3D measured data for comparison. First plot: d_{max}; second plot: 10 cm depth; third plot: 30 cm depth.

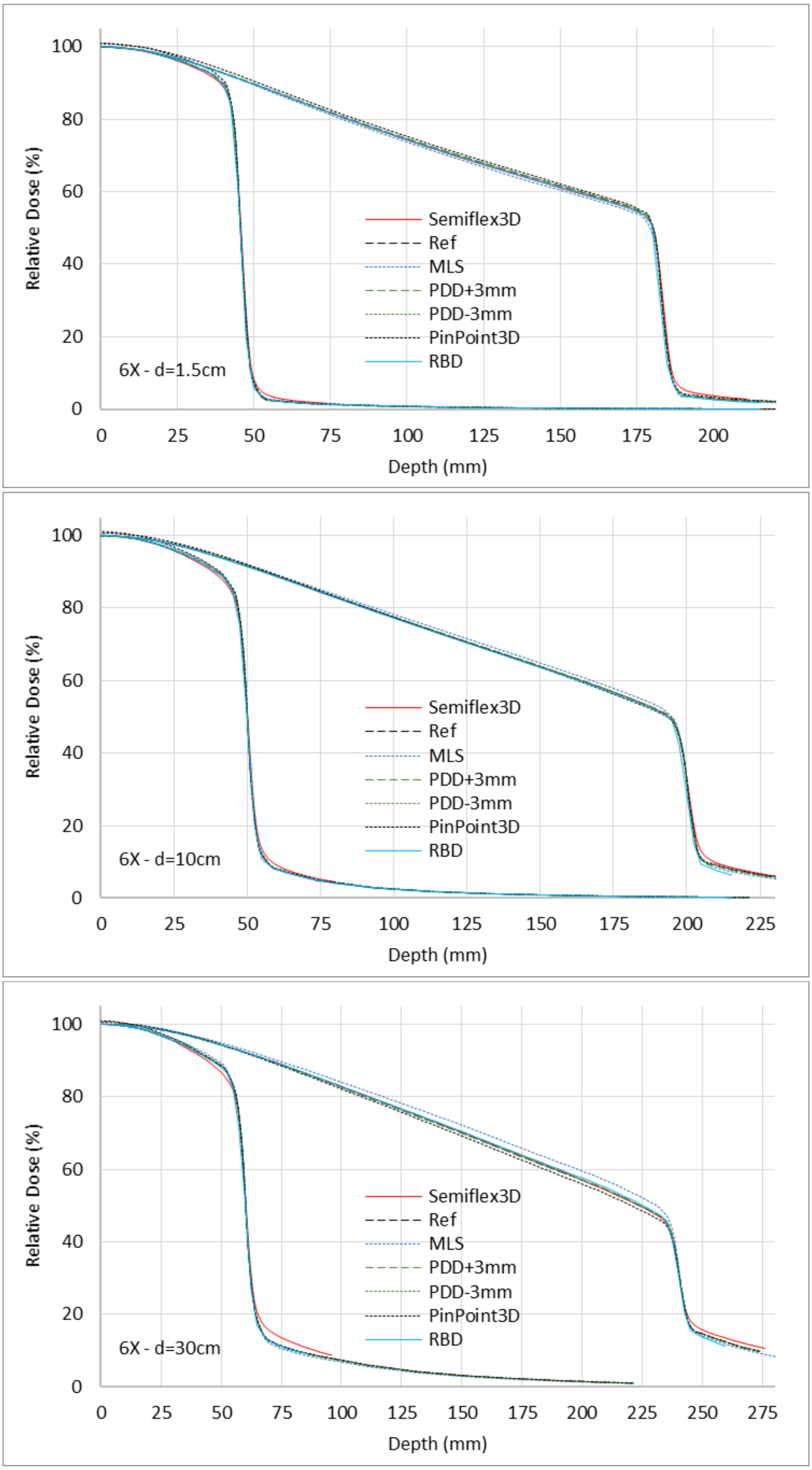

Supplement: Supplementary file 1 — Additional file 1. [file 13014_2020_1610_MOESM1_ESM.pdf]
